# Swedish prospective multicenter trial evaluating sentinel lymph node biopsy after neoadjuvant systemic therapy in clinically node-positive breast cancer

**DOI:** 10.1007/s10549-017-4164-1

**Published:** 2017-02-21

**Authors:** Linda Holmstrand Zetterlund, Jan Frisell, Athanasios Zouzos, Rimma Axelsson, Thomas Hatschek, Jana de Boniface, Fuat Celebioglu

**Affiliations:** 1 Department of Clinical Science and Education Södersjukhuset, Karolinska Institutet, Stockholm, Sweden; 2 Department of Surgery, Södersjukhuset, 118 83 Stockholm, Sweden; 3 Department of Molecular Medicine and Surgery, Karolinska Institutet, Stockholm, Sweden; 4 Departmnet of Breast and Endocrine Surgery, Karolinska University Hospital, 171 76 Stockholm, Sweden; 5 Department of Radiology, Södersjukhuset, 118 83 Stockholm, Sweden; 6 Department of Clinical Science, Intervention and Technology, Division of Radiography, Karolinska Institutet, Stockholm, Sweden; 7 Department of Radiology, Karolinska University Hospital Huddinge, 141 86 Stockholm, Sweden; 8 Department of Oncology, Karolinska Institutet, Cancer Center Karolinska and Karolinska University Hospital, 171 76 Stockholm, Sweden; 9 Department of Surgery, Capio St Göran’s Hospital, 112 81 Stockholm, Sweden

**Keywords:** Sentinel lymph node biopsy, Breast cancer, Neoadjuvant systemic therapy, False-negative rate, Identification rate, Node-positive

## Abstract

**Purpose:**

Patients with clinically node-positive breast cancer planned for neoadjuvant systemic therapy (NAST) may draw advantages from the nodal downstaging effect and reduce the extent of axillary surgery with sentinel lymph node biopsy (SLNB) performed after NAST. Since there are concerns about lower sentinel lymph node (SLN) detection and higher false-negative rates (FNR) in this setting, our aim was to define the accuracy of SLNB after NAST.

**Methods:**

This Swedish national multicenter trial prospectively recruited 195 breast cancer patients from ten hospitals with T1–T4d biopsy-proven node-positive disease planned for NAST between October 1, 2010 and December 31, 2015. Clinically node-negative axillary status after NAST was not mandatory. SLNB was always attempted and followed by a completion axillary lymph node dissection (ALND).

**Results:**

The SLN identification rate was 77.9% (152/195) but improved to 80.7% (138/171) with dual mapping. The median number of SLNs was two (range 1–5). A positive SLNB was found in 52% (79/152), almost 66% (52/79) of whom had additional positive non-sentinel lymph nodes. The overall pathologic nodal response rate was 33.3% (66/195). The overall FNR was 14.1% (13/92) but decreased to 4% (2/50) when only patients with two or more sentinel nodes were analyzed.

**Conclusions:**

In biopsy-proven node-positive breast cancer, SLNB after NAST is feasible even though the identification rate is lower than in clinically node-negative patients. Since the overall FNR is unacceptably high, the omission of ALND should only be considered if two or more SLNs are identified.

## Introduction

Sentinel lymph node biopsy (SLNB) is today the gold standard nodal staging procedure in clinically node-negative early-stage breast cancer. It is associated with improved staging accuracy and reduced arm morbidity compared with axillary lymph node dissection (ALND) [1, 2]. In approximately 70% of early-stage breast cancer patients, SLNB is negative, and ALND can safely be omitted [3]. The safety of SLNB in larger breast tumors has subsequently also been confirmed [4–6].

Locally advanced or inflammatory breast cancer (IBC) planned for neoadjuvant systemic therapy (NAST) implies an increased risk of dissemination to the regional lymph nodes at diagnosis. The standard nodal staging procedure is consequently ALND. Since the indications for NAST have expanded to also encompass operable breast cancer with aggressive tumor biology, the proportion of clinically node-positive patients planned for NAST has decreased [7]. Additionally, current NAST regimens in combination with targeted dual anti-HER2 therapies in HER2-positive patients can achieve nodal downstaging in as many as 70% of patients [8, 9]. As a consequence, SLNB was introduced in the neoadjuvant setting.

 While SLNB after NAST in clinically node-negative (cN0) patients at diagnosis is associated with high accuracy [10, 11], its use in clinically node-positive (cN1) patients is controversial owing to high false-negative rates [12, 13]. According to the ASCO guidelines from 2014, SLNB may be performed not only in cN0 patients with operable breast cancer but also in cN1 patients downstaged to clinical node negativity (ycN0) after NAST; the guidelines advise against performing SLNB in inflammatory breast cancer and do not support SLNB in locally advanced breast cancer due to insufficient data [14]. According to NCCN guidelines from 2016 (version 2.2016), the axilla may be restaged by SLNB after NAST in cN1 patients at diagnosis if the axilla becomes clinically node-negative after NAST (ycN0); however, marking the biopsied lymph nodes to secure their removal is recommended [15]. Dual mapping is advised to improve the false-negative rate (FNR), which is otherwise higher than 10% in this subgroup [16]. As the FNR correlates inversely with the number of sentinel lymph nodes retrieved, it is improved in those cases with two or more SLNs identified [17]. In addition, the identification rate (IR) after NAST is lower than for clinically node-negative patients at presentation [10, 18] but can be improved with dual mapping [19].

 This trial’s primary aim was to define the accuracy of SLNB after NAST in a multicenter setting in upfront clinically node-positive patients with T1–4d breast cancer.

## Methods

The present Swedish prospective multicenter trial recruited consecutive patients with biopsy-proven invasive T1–4d breast cancer planned for NAST from 20 invited hospitals of which 10 actively recruited patients between October 1, 2010 and December 31, 2015. Ultrasound of the axilla was performed, and if suspicious lymph nodes were encountered sonographically or by physical examination, fine-needle aspiration cytology (FNAC) was performed. Patients were enrolled into two arms depending on their axillary status at presentation.

 Clinically N1 patients with biopsy-proven axillary lymph node metastasis were eligible in the here presented arm of the trial which also covered patients with inflammatory breast cancer (IBC). After NAST, SLNB was attempted in all patients together with a completion ALND irrespective of the result of the SLNB. Clinical node negativity after NAST was not a requirement for SLNB to be attempted. Exclusion criteria were allergic reactions to blue dye or radiolabelled colloid, and inability to give informed consent.

Clinically N0 patients had SLNB performed before the start of NAST and will be reported separately.

For more details about the trial, see Clinical.Trials.gov identifier NCT02031042.

### Neoadjuvant therapy

Both neoadjuvant chemotherapy and endocrine therapy were eligible treatments. Standard chemotherapy regimens contained anthracyclines and taxanes, and were given either according to regional guidelines or within ongoing study protocols. Endocrine therapy consisted of aromatase inhibitors. Anti-HER2 therapy was given in combination with taxane-based chemotherapy. Altered or interrupted treatment was recorded together with the reason for disruption.

### Response evaluation

Clinical and radiological response was here evaluated by comparing findings in breast and axillary lymph nodes at diagnosis with those after termination of treatment before surgery. Clinical and radiological response was classified according to the UICC criteria [20] apart from radiological partial response which was classified according to the RECIST criteria as more than 30% decrease in tumor load measured on the greatest diameter [21]. Pathologic response was graded as described by Sataloff et al. evaluating tumor (T) and nodes (N) separately [22] as presented in Table 4.

Post-therapy stage classification (ypTNM) was based on definitions stated in the 7th edition of the AJCC staging system [23]. Pathologic complete response was defined as no residual invasive disease in the breast and axillary lymph nodes (ypT0/is ypN0). The presence of isolated tumor cells (ITC) [ypN0(i+)] was not considered nodal pCR [24].

### Lymphatic mapping technique

Preoperative lymphoscintigraphy was optional. Lymphatic mapping was performed with ^99m^Tc-labeled nanocolloid followed by peroperative use of gamma probe, Patent Blue V Dye, or both. The magnetic tracer superparamagnetic iron oxide was used alone or in combination with vital blue dye in a few cases. The definition of a sentinel lymph node was the hottest node, any node with more than 10% of the radioactivity of the hottest node, any blue node, or clinically suspicious nodes on surgical digital exploration.

### Pathologic assessment of lymph nodes

Lymph nodes were handled and assessed according to Swedish National Guidelines for Pathologists. Intraoperative frozen section analysis was not mandatory. All sentinel lymph nodes were fixed in formalin, sliced at 2 mm intervals, and embedded in paraffin. Each paraffin block was then sectioned at three 200 µm levels and each level stained with hematoxylin and eosin. Further staining with cytokeratin if no cancer cells were detected was not mandatory, and was not performed in non-sentinel nodes.

Sentinel lymph node (SLN) metastases were classified according to the 7th edition of the AJCC breast cancer staging manual [25].

### Surgery

Breast surgery was either breast-conserving surgery or mastectomy. All patients underwent SLNB and a completion axillary dissection of levels I and II.

### Definitions

Clinical tumor stage (cT) was based on pre-NAST radiological size measured by mammography or ultrasound.

The IR was defined as the number of patients with a successfully identified SLN divided by the total number of patients in whom an SLNB was attempted. The FNR was defined as the proportion of patients with a negative SLNB but at least one positive non-sentinel lymph node, divided by all patients with an identified SLNB and at least one positive lymph node after NAST. Accuracy was defined as the proportion of patients with a true-positive or true-negative SLNB out of all patients with successfully identified SLNs.

### Statistical analysis

Continuous variables are presented as median values with their ranges and categorical variables as distributions with their percentages. Comparison of groups according to sentinel lymph node status was performed after exploring normal data distribution. For comparison of non-parametric continuous data, the Mann–Whitney U test was applied. For comparison of non-parametric categorical data, Fisher’s exact test was used. A *p* value of <0.05 was considered statistically significant. Sample size calculation was only performed for the cN0 arm, which was the main purpose of this multicenter trial. The statistical software program IBM SPSS Statistics for Windows Version 23.0 (Armonk, NY, USA) was used for all analyzes.

## Results

### Patients

**Fig. 1:**
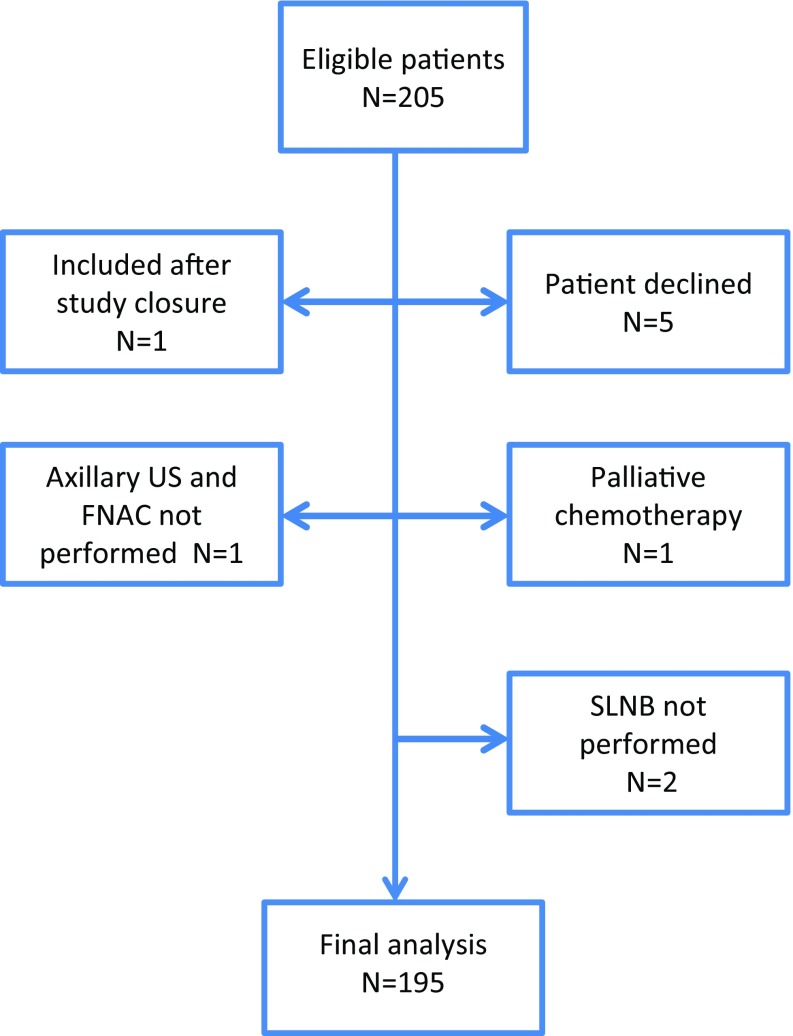
CONSORT diagram. US ultrasound, FNAC fine needle aspiration cytology, SLNB sentinel lymph node biopsy

Of 205 eligible patients, 195 patients from ten hospitals operated by 45 surgeons entered the final analysis. A CONSORT diagram is presented in Fig. 1. Median age was 50 years (range 27–84) and median radiological tumor size was 40 mm (range 11–160). Fifteen patients presented with inflammatory breast cancer (IBC), T4d. The axilla was examined by ultrasound at diagnosis in 99.0% (193/195) of patients, and in 98.9% (191/193), sonographically suspicious lymph nodes were identified. The two patients not examined by axillary ultrasound had suspicious lymph nodes on physical examination. All patients had cytologically confirmed node-positive disease before the initiation of NAST. Clinicopathologic and treatment characteristics are presented in Table 1.

**Table 1:** Clinicopathologic and treatment characteristics

	No. (%)
No. of patients	195
Median years, age	50, range 27–84
T-stage at presentation	
T1	25 (12.8)
T2	94 (48.2)
T3	61 (31.3)
T4d (inflammatory)	15 (7.7)
Histological type	
Ductal	158 (83.6)
Lobular	14 (7.4)
Other	17 (9.0)
Unknown	6 (3.1)
Nottingham histological grade	
I	1 (0.7)
II	79 (55.6)
III	62 (43.7)
Unknown	53 (27.2)
ER-positive	134 (68.7)
PR-positive	95 (48.7)
HER2-positive	62 (31.8)
Neoadjuvant systemic therapy	
Anthracycline plus taxane	184 (94.4)
Anthracycline only	7 (3.6)
Other	3 (1.5)
Aromatase inhibitor	1 (0.5)

*T*-stage tumor size radiologically, *ER* estrogen receptor, *PR* progesteron receptor, *HER2* human epidermal growth factor receptor 2

### Treatment

All but one patient received neoadjuvant chemotherapy. The majority had anthracycline plus taxane-based regimens (94.4%; 184/195). Only one patient in 195 received neoadjuvant endocrine therapy (0.5%). Neoadjuvant systemic therapy regimens are presented in Table 1.

In twelve patients, treatment was interrupted due to intolerable side effects (7), toxicity (2), lack of response (2), or unknown reason (1). In 35 patients, treatment was altered due to intolerable side effects (19), toxicity (9), and lack of response (5); in two patients, a planned shift of therapy was not carried out. Of all HER2-positive patients, 93.5% (58/62) received targeted treatment, 33.9% (19/56) of whom received both trastuzumab and pertuzumab. Breast-conserving surgery was performed in 51 of 195 patients (26.2%).

### SLN detection after NAC

Lymphatic mapping was performed using a combination of radioisotope and Patent blue dye in 87.5% (168/192), isotope alone in 5.2% (10/192), and blue dye alone in 3.6% (7/192) of patients. In 3.6% (7/192), magnetic tracer alone or in combination with blue dye was used.

Overall, at least one SLN was identified in 152 of 195 patients yielding an IR of 77.9%. With dual mapping, regardless of method, the IR was 80.7% (138/171).

After excluding patients with IBC, the IR was 79.4% (143/180), and if dual mapping was employed, it further improved to 82.8% (130/157).

The median number of retrieved SLNs was two (range 1–5). In 52% (79/152), SLNB was positive, and in 88.6% (70/79) of these, at least one macrometastasis was found. The median number of harvested additional axillary lymph nodes was 11 (range 3–41). Fifty-two of 79 (65.8%) SLNB-positive patients had additional non-sentinel positive lymph nodes. Of all 195 patients, 124 (63.6%) had residual axillary tumor burden after NAST in either SLNs and/or non-sentinel lymph nodes. The median number of positive nodes was three (range 1–29).

### False-negative rate

Thirteen patients had a negative SLNB after NAST but at least one positive lymph node in non-sentinel lymph nodes yielding an overall FNR of 14.1% (13/92). A comparison of lymph node status in SLNs and non-sentinel lymph nodes is presented in Table 2. Among the false-negative cases, the median number of positive lymph nodes was 1 (range 1–9) and the median number of retrieved axillary lymph nodes including sentinel lymph nodes was 12 (range 5–20), see Table 3. There were two patients with IBC, and the FNR after excluding these patients was 12.6% (11/87). False-negative rates calculated for different scenarios are presented in Table 4.

**Table 2:** Comparison of lymph node status in SLNs and overall axillary lymph node status after NAST

SLNB	Overall axillary nodal status (SLNB and ALND)		
	Positive	Negative	Total
Positive	79	0	79
Negative	13	60	73
Total	92	60	152

Sensitivity 85.9% (79/92), specificity 100.0% (60/60), positive predictive value 100.0% (79/79), negative predictive value 82.2% (60/73)

*SLN* sentinel lymph node, *SLNB* sentinel lymph node biopsy, *NAST* neaodjuvant systemic therapy, *ALND* axillary lymph node dissection

**Table 3:** Thirteen patients with false negative SLNs and corrresponding non-sentinel lymph nodes

Patient number	IBC	Number of SLNs	Lymph node status in SLNs	Number of non-sentinel lymph nodes	Lymph node status non-sentinel nodes	Total number of axillary lymph nodes
33	No	2	ypN0	7	ypN1mi	9
39	No	1	ypN0	9	ypN1mi	10
103	No	1	ypN0	5	ypN1	6
202	No	1	ypN0	11	ypN1(3)	12
226	Yes	1	ypN0	15	ypN1mi(7)	16
229	No	1	ypN1(i+)	11	ypN1	12
232	No	1	ypN0	19	ypN1(9)	20
236	No	1	ypN0	4	ypN1	5
292	No	1	ypN0	11	ypN1	12
294	No	2	ypN1(i+)	6	ypN1mi	8
392	No	1	ypN0	14	ypN1	15
442	Yes	1	ypN0	14	ypN1(6)	15
450	No	1	ypN1(i+)	14	ypN1	15

*SLNs* sentinel lymph nodes, *NAST* neoadjuvant systemic therapy, *ypN1* macrometastasis, *ypN1mi* micrometastasis, *ypN1*(*i*+) isolated tumor cells, *IBC* inflammatory breast cancer

**Table 4:** False negative SLN findings after NAST in different scenarios

Scenario	True pos (*n*)	False neg (*n*)	FNR^a^ (%)
Overall	79	13	14.1
Dual mapping performed	71	11	13.4
IBC excluded (*n* = 15)	76	11	12.6
ITC considered ypN+	87	10	10.3
SLNB with 1 node retrieved	31	11	26.2
SLNB with ≥2 nodes	48	2	4.0
SLNB with ≥3 nodes	23	0	0.0

^a^Calculated as the number of patients with a false negative SLN in each scenario divided by the number of false negative and true positive SLNs in the same scenario

*NAST* neoadjuvant systemic therapy, *FNR* false negative rate, *SLN* sentinel lymph node, *SLNB* sentinel lymph node biopsy, *IBC* inflammatory breast cancer, *ITC* isolated tumor cells, *FNR* false negative rate

There was no significant difference between patients with a false-negative compared to a true-positive or true-negative SLNB regarding age distribution, tumor size, grade or type, hormone receptor status, HER2 positivity, breast surgery performed, neoadjuvant therapy regimen, anti-HER2-targeted therapy, or proportion of patients with interrupted NAST. However, patients with false-negative SLNs had significantly more altered chemotherapy regimens (46.2%; 6/13) compared with patients with a true-positive or true-negative SLNB (15.2%; 21/138; *p* = 0.013).

### Response evaluation

There was no statistical difference in clinical or radiological response in neither tumor nor lymph nodes between patients with a true-positive or true-negative compared to a false-negative SLNB result. However, there were significantly more patients with a complete/near-complete pathologic response in the tumor (Sataloff A) in the true-positive/true-negative group (35.3%) than in the false-negative group (7.7%; *p* = 0.044), see Table 5.

**Table 5:** Comparison of response between patients with false-negative to true-positive and true-negative SLNs after NAST

	True-pos and true-neg (%)	False-neg (%)	*P*
No. of patients	139	13	
Pathologic response, tumoral (ypT)			
Sataloff T-A	49 (35.3)	1 (7.7)	
Sataloff T-B	39 (28.1)	7 (53.8)	
Sataloff T-C	43 (30.9)	3 (23.1)	
Sataloff T-D	8 (5.8)	2 (15.4)	0.044
Pathological response, nodal (ypN)			
Sataloff N-A	38 (27.3)	0 (0.0)	
Sataloff N-B	22 (15.8)	0 (0.0)	
Sataloff N–C	39 (28.1)	5 (28.5)	
Sataloff N-D	40 (28.8)	8 (61.5)	0.010

Sataloff T-A: Total or near total therapeutic effect; Sataloff T-B: >50% therapeutic effect but less than total or near total; Sataloff T-C: <50% therapeutic effect, but effect evident; Sataloff T-D: No therapeutic effect

Sataloff N-A: Evidence of therapeutic effect, no metastatic disease; Sataloff N-B: No nodal metastasis or therapeutic effect; Sataloff N-C: Evidence of therapeutic effect but nodal metastasis still present; Sataloff N-D: Viable metastatic disease, no therapeutic effect

*SLN* sentinel lymph node, *NAST* neoadjuvant systemic therapy

Of all patients with an identified SLNB after NAST, 32.9% (50/152) had a complete pathologic response in the breast (ypT0/is), 36.2% (55/152) a complete pathologic nodal response (ypN0), and 27.6% (42/152) had an overall complete pathologic response (ypCR). The corresponding figures for all 195 patients were 30.8% (60/195), 33.3% (65/195), and 25.6% (50/195), respectively.

## Discussion

This Swedish multicenter trial evaluates the accuracy of SLNB in the neoadjuvant setting. In the present part of the trial, SLNB was attempted after NAST together with concomitant ALND in 195 T1–4d breast cancer patients with biopsy-proven lymph node metastasis at diagnosis. The results of the other part of the same trial regarding cN0 patients in whom SLNB was performed before and ALND after NAST are reported separately.

The performance of SLNB after NAST in cN0 patients at diagnosis is associated with lower IR and higher FNR than SLNB upfront [10, 18]. The assumed causes for these findings are fibrosis of the lymphatic channels after NAST, altering lymphatic drainage patterns and differential eradication of disease in sentinel and non-sentinel lymph nodes [26, 27]. In patients with cN1 disease, SLNB after NAST has been questioned because of unacceptably high FNR in some earlier reports [12–14].

The overall IR in the present cohort was 77.9% (152/195). Excluding 15 patients with IBC improved the IR marginally. According to our knowledge, there are only two papers addressing the accuracy of SLNB after NAST in IBC. Both conclude that the method is unreliable in IBC, but base their conclusions on only eight and 20 patients, respectively [28, 29]. The overall FNR, too, improved in our trial after excluding IBC from analysis. ASCO guidelines from 2014 discourage the performance of SLNB in IBC also after NAST. In case of locally advanced breast cancer downstaged by NAST, data were still considered insufficient to recommend SLNB after NAST [14]. Based on these small studies, we conclude that SLNB after NAST in IBC is feasible but less accurate compared with locally advanced or operable breast cancer, but larger prospective studies are warranted.

An important measure to improve not only the IR but also the FNR is dual mapping, which was recommended but not mandatory in our trial. Dual mapping yielded better overall IR, which is consistent with the prospective SENTINA study reporting an IR of 80.1% [13]. Also in the NSABP B-27 trial and in ACOSOG Z1071 trial, mapping with radioisotope only or in combination with blue dye was more successful than blue dye alone [19, 27]. In the latter trial, the FNR was significantly reduced by the use of dual mapping [12]. Thus, dual mapping should be the method of choice also in the neoadjuvant setting.

The overall FNR in our trial correlates with the pooled estimate of 15.1% in a systematic review and meta-analysis evaluating SLNB after NAST in patients with pathologically confirmed node-positive breast cancer. Nijnatten et al. performed a subgroup analysis in which FNR was significantly lower when two or more SLNs were removed [17]. In the ACOSOG Z1071 trial, the FNR decreased from 21.1 to 9.1% when three or more nodes were examined instead of two [12]. In arm C of the four-armed prospective SENTINA study, the FNR decreased from 24.3% if one to 18.5% if two nodes were removed [13]. Also in our trial, the FNR decreased dramatically when two or more SLNs were retrieved.

A limitation of our trial was that clinical restaging after completion of NAST was not performed. In the ACOSOG Z1071 trial, patients were restaged with axillary ultrasound after NAST. Although the reduction of FNR was not statistically significant, sonographically normal lymph nodes correlate with a decreased likelihood of residual nodal disease which can help selecting patients for SLNB after NAST [30].

If all patients with ITC in the SLNs were classified as ypN1 in our study, the overall FNR would have decreased to 10.3%. Since IHC staining was not mandatory, unlike in the SN FNAC study, undiagnosed ITCs are likely. In the SN FNAC study, FNR decreased from 13.3% when ITC was considered ypN0 to only 8.4% when SN metastases of any size were considered positive [31].

In this trial, there was no significant difference in clinical and radiological response between patients with a false-negative compared with a true-positive or true-negative SLN result. The correlation between clinical and pathological response is not reliable related to both the primary tumor and regional lymph nodes [27]. Galimberti et al. retrospectively evaluated 396 cT1–4 cN0/cN1/2 patients who remained or became ycN0 after NAST. After five years of follow-up, overall survival was not significantly worse in the cN1/2 group. SLN negativity after NAST was a significant predictor of good outcome but only if the breast tumor had responded well [32].

## Conclusion

This trial confirms the feasibility of SLNB after NAST in biopsy-proven node-positive breast cancer at diagnosis. The IR, however, is lower compared with clinically node-negative patients and the overall FNR is unacceptably high if only one SLN is retrieved. To optimize both IR and FNR, dual mapping should be the method of choice. If only one SLN can be accurately identified and retrieved, a completion ALND should be considered. Pre-NAST marking of the cytologically verified lymph node, selecting only patients with sonographically unsuspicious lymph nodes for SLNB after NAST, and broadening the definition of SLN metastasis after NAST to include isolated tumor cells, all have the potential of further decreasing the FNR.
